# FABP1 expression in human tumors: a tissue microarray study on 17,071 tumors

**DOI:** 10.1007/s00428-022-03394-5

**Published:** 2022-08-11

**Authors:** David Dum, Ana Ocokoljic, Maximilian Lennartz, Claudia Hube-Magg, Viktor Reiswich, Doris Höflmayer, Frank Jacobsen, Christian Bernreuther, Patrick Lebok, Guido Sauter, Andreas M. Luebke, Eike Burandt, Andreas H. Marx, Ronald Simon, Till S. Clauditz, Sarah Minner, Anne Menz, Franziska Büscheck, Natalia Gorbokon, Stefan Steurer, Niclas C. Blessin, Till Krech

**Affiliations:** 1 Institute of Pathology, University Medical Center Hamburg-Eppendorf, Martinistr. 52, 20246 Hamburg, Germany; 2 Institute of Pathology, Clinical Center Osnabrueck, Osnabrueck, Germany; 3 Department of Pathology, Academic Hospital Fuerth, Fuerth, Germany

**Keywords:** FABP1, Tissue microarray, Immunohistochemistry, Diagnostic, Human cancer

## Abstract

Fatty acid–binding proteins (FABPs) play a pivotal role in the metabolism of fatty acids and are expressed in a tissue-specific manner. FABP1 is most abundantly expressed in the liver where it accounts for about 10% of the total cytosolic protein and is thought to have diagnostic utility. To comprehensively determine FABP1 expression in normal and neoplastic tissues, a tissue microarray containing 17,071 samples from 150 different tumor types and subtypes as well as 608 samples of 76 different normal tissue types was analyzed by immunohistochemistry. Among normal tissues, a strong FABP1 immunostaining was observed in hepatocytes, proximal tubuli of the kidney and epithelium of small intestine, appendix, and the colorectum. FABP1 positivity was found in 24 of 150 tumor categories, including 17 tumor categories with at least 1 strongly positive case. The highest FABP1 positivity rates were seen in colorectal adenomas (86%), in colorectal adenocarcinomas (71.1%), and in hepatocellular carcinomas (65.3%), followed by mucinous carcinoma of the ovary (34.6%), cholangiocarcinoma (21.6%), and various adenocarcinomas from the digestive tract (10–23%). Eleven additional entities had positivity rates between 0.2 and 6.5%. FABP1 staining was not seen in 169 primary adenocarcinomas of the lung. In colorectal cancer, reduced FABP1 expression was linked to poor-grade, right-sided tumor location, microsatellite instability (*p* < 0.0001 each), and absence of BRAF V600E mutations (*p* = 0.001), but unrelated to pT and pN status. FABP1 expression has considerably high tumor specificity. As FABP1 expression was virtually absent in adenocarcinomas of the lung, FABP1 immunohistochemistry might be particularly helpful to assist in the identification of metastatic colorectal or gastrointestinal adenocarcinoma to the lung.

**Supplementary Information:**

The online version contains supplementary material available at 10.1007/s00428-022-03394-5.

## Introduction 

Fatty acid–binding proteins (FABPs) constitute a family of at least 9 proteins, which play a pivotal role in the metabolism of fatty acids and related molecules. All FABPs are expressed in a tissue-specific manner, and their levels of expression are considered to be proportional to the rate of fatty acid metabolism [1–4]. Fatty acid–binding protein 1, also termed liver FABP (L-FABP), is expressed from the FABP1 gene located at human chromosome 2p11.2 [5]. The 14-kilodalton protein is most abundantly expressed in the liver where it accounts for about 10% of the total cytosolic protein [6, 7]. FABP1 is involved in the binding, transport, and metabolism of long-chain fatty acids in the liver [6, 7]. Unlike other members of the FABP family, the large hydrophobic binding pocket located in the FABP1 structure is capable of binding to a particularly broad spectrum of hydrophobic ligands and to simultaneously attach multiple ligands [8]. FABP1 ligands include bilirubin, bile acids, or monoglycerides but also benzodiazepines, fibrates, β-blockers, and non-steroidal anti-inflammatory drugs [9, 10]. FABP1 plays a significant role in preventing cytotoxicity/activity of these molecules [9]. Several mutations of the FABP1 gene have been linked to specific metabolic conditions including obesity, cardiovascular disease, and diabetes [8, 11].

Because of its high tissue specificity, FABP1 expression analysis by immunohistochemistry might have diagnostic utility. Studies using FABP1 immunohistochemistry have so far described FABP1 positivity in 47–100% of hepatocellular carcinomas [12, 13], 47.4–83.3% of various subtypes of lung cancer [14], 30–81.5% of colorectal carcinomas [15, 16], 38.6% of gastric adenocarcinomas [17], 27–36.4% of various kidney cancer subtypes [18], and in 12.1% of pancreatic carcinomas [19]. Many other tumor entities have so far not been systematically analyzed.

In order to comprehensively assess the potential diagnostic utility of FABP1 expression in cancer, a preexisting set of tissue microarrays containing more than 17,000 tumor tissue samples from 150 different tumor types and subtypes as well as 76 non-neoplastic tissue categories was analyzed by immunohistochemistry (IHC) in this study.

## Material and methods

### Tissue microarrays (TMAs)

The normal TMA was composed of 8 samples from 8 different donors for each of 76 different normal tissue types (608 samples on one slide). The cancer TMAs contained a total of 17,071 primary tumors from 150 tumor types and subtypes. Detailed histopathological data on tumor phenotype and molecular data on microsatellite instability, RAS mutations, and BRAF V600E mutations were available from the majority of 2351 colorectal adenocarcinomas. The composition of both normal and cancer TMAs is described in detail in the “Results” section. All samples were from the archives of the Institute of Pathology, University Hospital of Hamburg, Germany; the Institute of Pathology, Clinical Center Osnabrück, Germany; and the Department of Pathology, Academic Hospital Fuerth, Germany. Tissues were fixed in 4% buffered formalin and then embedded in paraffin. The TMA manufacturing process was described earlier in detail [20, 21]. In brief, one tissue spot (diameter: 0.6 mm) was transmitted from a cancer containing donor block in an empty recipient paraffin block. The use of archived remnants of diagnostic tissues for manufacturing of TMAs and their analysis for research purposes as well as patient data analysis has been approved by local laws (HmbKHG, §12) and by the local ethics committee (Ethics Commission Hamburg, WF-049/09). All work has been carried out in compliance with the Helsinki Declaration.

### Immunohistochemistry (IHC)

Freshly prepared TMA sections were immunostained on one day in one experiment. Slides were deparaffinized with xylol, rehydrated through a graded alcohol series, and exposed to heat-induced antigen retrieval for 5 min in an autoclave at 121 °C in pH 7,8 Dako target Retrieval Solution™ (Agilent, CA, USA; #S2367). Endogenous peroxidase activity was blocked with Dako Peroxidase Blocking Solution™ (Agilent, CA, USA; #52,023) for 10 min. Primary antibody specific against FABP1 protein (mouse monoclonal, MSVA-501 M, #3737-501 M, MS Validated Antibodies, Hamburg, Germany) was applied at 37 °C for 60 min at a dilution of 1:150. Bound antibody was visualized using the EnVision Kit™ (Agilent, CA, USA; #K5007) according to the manufacturer’s directions. The sections were counterstained with haemalaun. For tumor tissues, the percentage of FABP1-positive tumor cells was estimated, and the staining intensity was semi-quantitatively recorded (0, 1 + , 2 + , 3 +). For statistical analyses, the staining results were categorized into four groups as described before [22]: negative, no staining at all; weak staining, staining intensity of 1 + in ≤ 70% or staining intensity of 2 + in ≤ 30% of tumor cells; moderate staining, staining intensity of 1 + in > 70%, or staining intensity of 2 + in > 30% but in ≤ 70% or staining intensity of 3 + in ≤ 30% of tumor cells; and strong staining, staining intensity of 2 + in > 70% or staining intensity of 3 + in > 30% of tumor cells. Examples of tumors with different scores are shown in Suppl. Figure 1.

### Statistics

Statistical calculations were performed with JMP 14 software (SAS Institute Inc., NC, USA). Contingency tables and the chi^2^ test were performed to search for associations between FABP1 immunostaining and tumor phenotype. A *p* value of ≤ 0.05 was defined as significant. Cox proportional hazard regression analysis was performed to test the statistical independence of associations between pathological and molecular variables.

## Results

### FABP1 in normal tissues

**Fig. 1:**
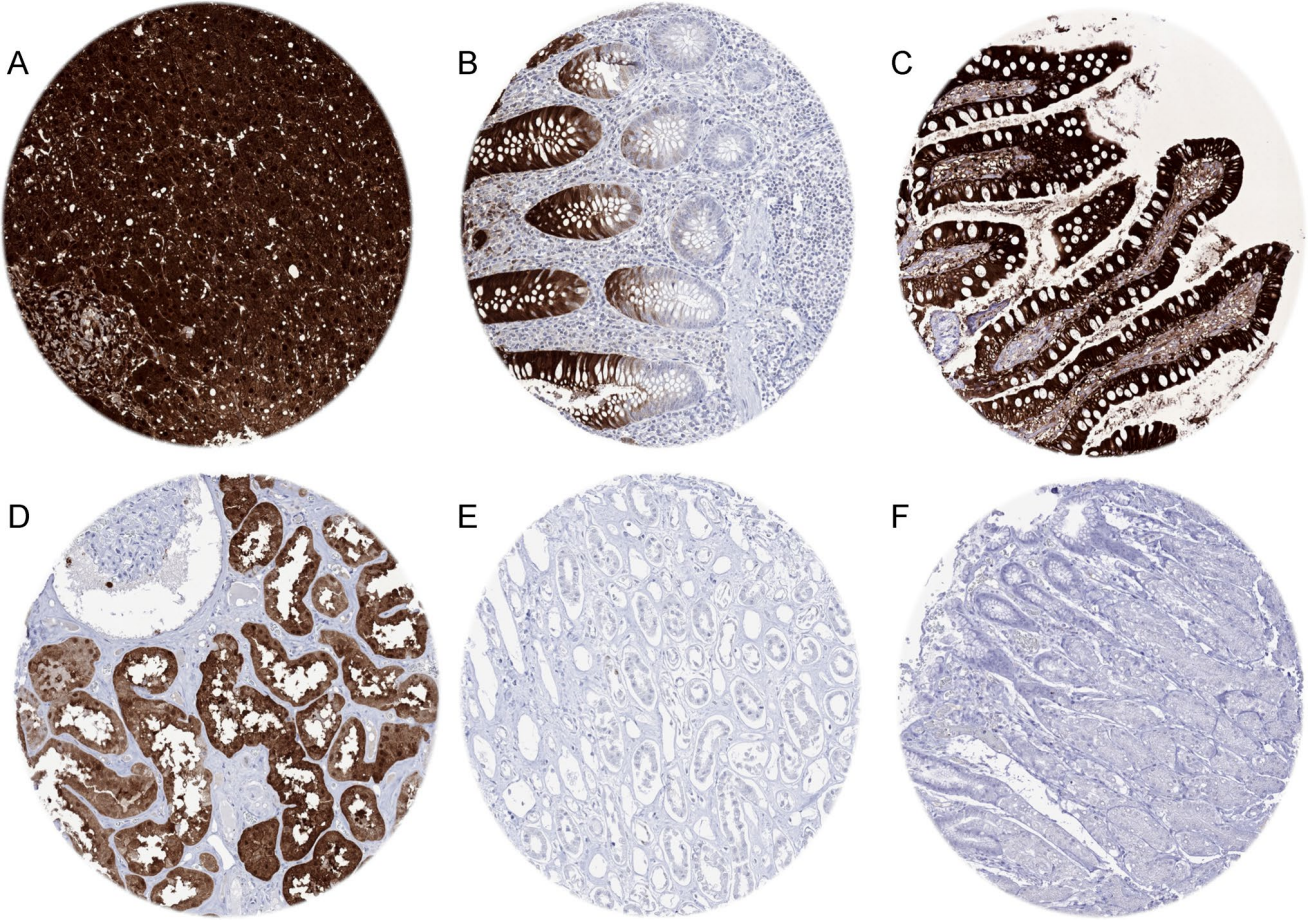
FABP1 immunostaining in normal tissues. The panels show a strong (3 +) cytoplasmic FABP1 staining of hepatocytes in the liver (**A**), surface epithelium of the appendix (**B**), and the ileum (**C**) as well as in proximal tubular cells of the kidney (**D**). FABP1 expression can be so strong in these tissues that considerable contamination artifacts occur in adjacent cells/tissues (**A**–**C**). FABP1 staining is lacking in the renal medulla (**E**) and in the stomach epithelium (**F**)

A strong FABP1 immunostaining was observed in hepatocytes of the liver, in proximal tubular cells of the kidney, and in epithelial cells of the small intestine, appendix, and the colorectum. In the entire intestine, the staining was strongest in the surface epithelium and sometimes low or even inexistent in the crypt bases. In the stomach epithelium, FABP1 staining was usually absent. Focal positivity was seen, however, in case of intestinal metaplasia. In case of very strong staining of intestinal or liver cells, adjacent structures often also showed FABP1 immunostaining. This is considered a contamination artifact due to diffusion of the antigen. Representative images of FABP1-positive normal tissues are shown in Fig. 1.

### FABP1 in cancer

**Fig. 2:**
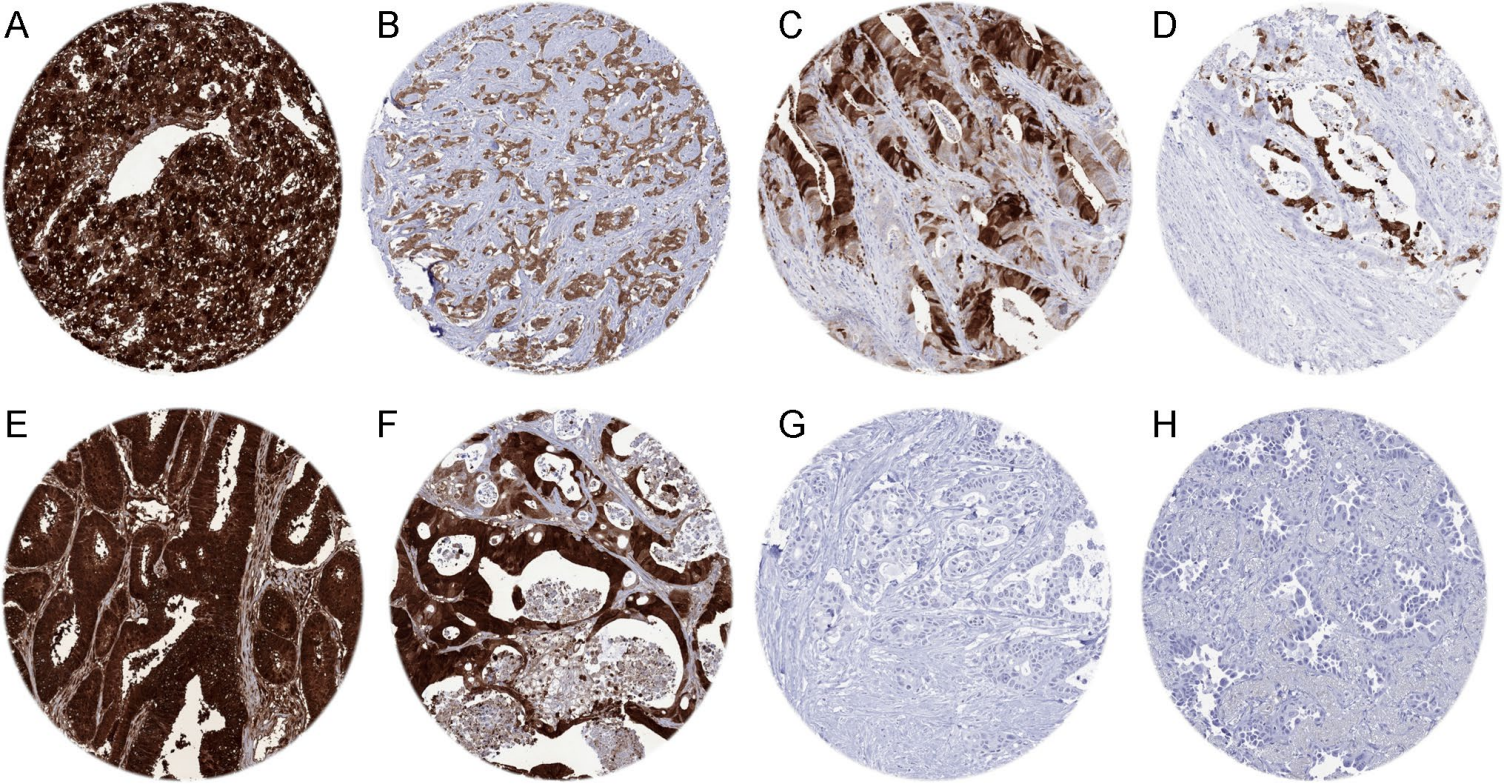
FABP1 immunostaining in cancer. The panels show a cytoplasmic FABP1 immunostaining of variable intensity in samples from hepatocellular carcinoma (**A**), cholangiocarcinoma (**B**), gastric adenocarcinoma (**C**), esophageal adenocarcinoma (**D**), colorectal adenocarcinoma (**E**), and an adenocarcinoma of the papilla of Vater (**F**). In several samples, FABP1 expression is so high that contamination artifacts occur in adjacent cells/tissues. FABP1 staining is completely absent in samples from a ductal adenocarcinoma of the pancreas (**G**) and an adenocarcinoma of the lung (**H**)

**Fig. 3:**
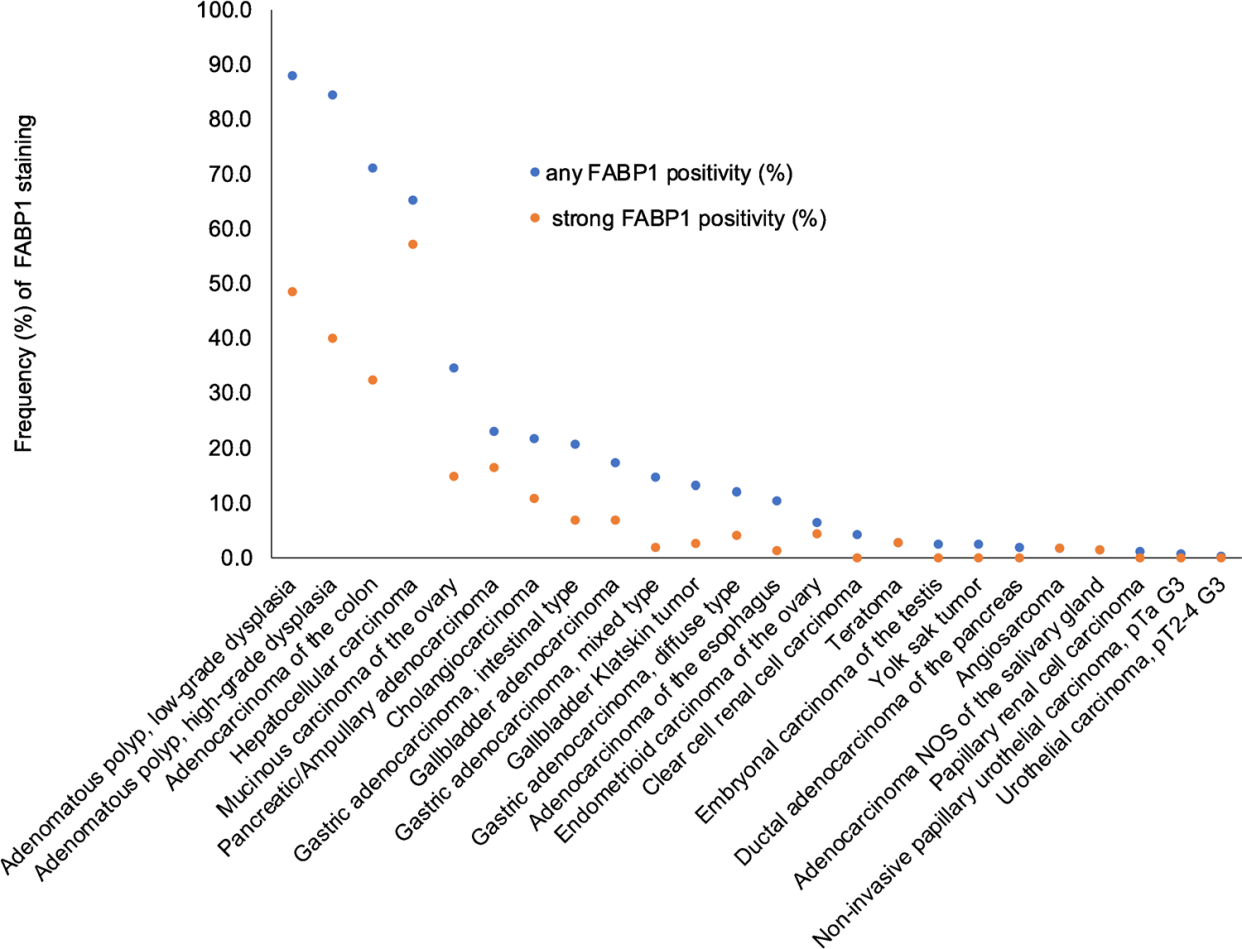
Ranking order of FABP1 immunostaining in tumors. Both the frequency of positive cases (blue dots) and the frequency of strongly positive cases (orange dots) are shown

A positive FABP1 immunostaining was detectable in 1980 (14%) of the 14,597 analyzable tumors, including 470 (3.2%) with weak, 563 (3.9%) with moderate, and 947 (6.5%) with strong immunostaining. Overall, 24 (16%) of 150 tumor categories showed detectable FABP1 expression with 17 (11%) tumor categories including at least one case with strong positivity (Table 1). Representative images of FABP1-positive tumors are shown in Fig. 2. By far the highest positivity rates were seen in colorectal adenomas (44–88%), in colorectal adenocarcinomas (71%), and in hepatocellular carcinomas (65%), followed by mucinous carcinoma of the ovary (35%), cholangiocarcinoma (22%), and various adenocarcinomas from the digestive tract (10–23%). Of note, none of our FABP1-positive cholangiocarcinomas qualified for a diagnosis of combined HCC-cholangiocarcinoma as all of these tumors showed a predominantly small-glandular growth pattern and did not show any HepPar1 or arginase1 immunostaining (data not shown). Eleven further tumor entities had positivity rates between 0.2 and 6.5%. A graphical representation of a ranking order of tumor entities according to their rate of FABP1-positive and strongly positive cases is given in Fig. 3. FABP1 expression was not found in any of 252 arrayed lung cancers, including 169 adenocarcinomas of the lung. FABP1 was also negative in all 85 pulmonary adenocarcinomas for which data were available from previous studies on CK20 [23], villin [24], and SATB2 [25]. Evidence for a possible enteric/intestinal differentiation had been found in 20 (24%) of these tumors because of a positive staining for at least one of these intestinal markers (Supplementary Table 1). The relationship between FABP1 immunostaining and histopathological and molecular features of colorectal adenocarcinomas and hepatocellular carcinomas are shown in Table 2. In colorectal cancer, reduced FABP1 expression was strikingly linked to histologic grade, microsatellite instability (MSI), and tumor location in the right side of the colon (*p* < 0.0001 each), and absence of BRAF V600E mutations (*p* = 0.001) but was unrelated to pT and pN status or RAS mutation status. A multivariate analysis including MSI, pT, pN, and histologic grade showed that associations between these parameters and reduced FABP1 expression was driven by the histologic grade and stage (*p* ≤ 0.05; Supplementary Table 2). Within 84 MSI tumors, reduced FABP1 expression was weakly associated with L0 status (*p* = 0.0203) and tumor location in the right colon (*p* = 0.0023). Within 1067 MSS tumors, reduced FABP1 expression was weakly associated with right-sided tumor location (*p* = 0.0372). In hepatocellular carcinomas, reduced FABP1 expression was linked to advanced stage (*p* = 0.0002), presence of lymph node metastasis (*p* = 0.0042), and female gender (*p* = 0.0002).

**Table 1:** FABP1 immunostaining in human tumors

			FABP1 immunostaining result				
	Tumor entity	On TMA (*n*)	Analyzable (*n*)	Negative (%)	Weak (%)	Moderate (%)	Strong (%)
Tumors of the skin	Pilomatrixoma	35	27	100.0	0.0	0.0	0.0
	Basal cell carcinoma	88	78	100.0	0.0	0.0	0.0
	Benign nevus	29	26	100.0	0.0	0.0	0.0
	Squamous cell carcinoma of the skin	90	89	100.0	0.0	0.0	0.0
	Malignant melanoma	46	44	100.0	0.0	0.0	0.0
	Malignant melanoma Lymph node metastasis	86	85	100.0	0.0	0.0	0.0
	Merkel cell carcinoma	46	35	100.0	0.0	0.0	0.0
Tumors of the head and neck	Squamous cell carcinoma of the larynx	110	80	100.0	0.0	0.0	0.0
	Squamous cell carcinoma of the pharynx	60	59	100.0	0.0	0.0	0.0
	Oral squamous cell carcinoma (floor of the mouth)	130	112	100.0	0.0	0.0	0.0
	Pleomorphic adenoma of the parotid gland	50	31	100.0	0.0	0.0	0.0
	Warthin tumor of the parotid gland	104	81	100.0	0.0	0.0	0.0
	Adenocarcinoma, NOS (Papillary Cystadenocarcinoma)	14	12	100.0	0.0	0.0	0.0
	Salivary duct carcinoma	15	10	100.0	0.0	0.0	0.0
	Acinic cell carcinoma of the salivary gland	181	129	100.0	0.0	0.0	0.0
	Adenocarcinoma NOS of the salivary gland	109	68	98.5	0.0	0.0	1.5
	Adenoid cystic carcinoma of the salivary gland	180	85	100.0	0.0	0.0	0.0
	Basal cell adenocarcinoma of the salivary gland	25	19	100.0	0.0	0.0	0.0
	Basal cell adenoma of the salivary gland	101	77	100.0	0.0	0.0	0.0
	Epithelial-myoepithelial carcinoma of the salivary gland	53	50	100.0	0.0	0.0	0.0
	Mucoepidermoid carcinoma of the salivary gland	343	243	100.0	0.0	0.0	0.0
	Myoepithelial carcinoma of the salivary gland	21	18	100.0	0.0	0.0	0.0
	Myoepithelioma of the salivary gland	11	10	100.0	0.0	0.0	0.0
	Oncocytic carcinoma of the salivary gland	12	8	100.0	0.0	0.0	0.0
	Polymorphous adenocarcinoma, low grade, of the salivary gland	41	32	100.0	0.0	0.0	0.0
	Pleomorphic adenoma of the salivary gland	53	40	100.0	0.0	0.0	0.0
Tumors of the lung, pleura, and thymus	Adenocarcinoma of the lung	196	169	100.0	0.0	0.0	0.0
	Squamous cell carcinoma of the lung	80	71	100.0	0.0	0.0	0.0
	Small cell carcinoma of the lung	16	12	100.0	0.0	0.0	0.0
	Mesothelioma, epithelioid	39	29	100.0	0.0	0.0	0.0
	Mesothelioma, other types	76	51	100.0	0.0	0.0	0.0
	Thymoma	29	24	100.0	0.0	0.0	0.0
Tumors of the female genital tract	Squamous cell carcinoma of the vagina	78	46	100.0	0.0	0.0	0.0
	Squamous cell carcinoma of the vulva	130	109	100.0	0.0	0.0	0.0
	Squamous cell carcinoma of the cervix	129	109	100.0	0.0	0.0	0.0
	Adenocarcinoma of the cervix	21	21	100.0	0.0	0.0	0.0
	Endometrioid endometrial carcinoma	236	207	100.0	0.0	0.0	0.0
	Endometrial serous carcinoma	82	56	100.0	0.0	0.0	0.0
	Carcinosarcoma of the uterus	48	42	100.0	0.0	0.0	0.0
	Endometrial carcinoma, high grade, G3	13	12	100.0	0.0	0.0	0.0
	Endometrial clear cell carcinoma	8	7	100.0	0.0	0.0	0.0
	Endometrioid carcinoma of the ovary	110	93	93.5	1.1	1.1	4.3
	Serous carcinoma of the ovary	559	510	100.0	0.0	0.0	0.0
	Mucinous carcinoma of the ovary	96	81	65.4	8.6	11.1	14.8
	Clear cell carcinoma of the ovary	50	47	100.0	0.0	0.0	0.0
	Carcinosarcoma of the ovary	47	39	100.0	0.0	0.0	0.0
	Granulosa cell tumor of the ovary	37	35	100.0	0.0	0.0	0.0
	Leydig cell tumor of the ovary	4	4	100.0	0.0	0.0	0.0
	Sertoli cell tumor of the ovary	1	1	100.0	0.0	0.0	0.0
	Sertoli-Leydig cell tumor of the ovary	3	3	100.0	0.0	0.0	0.0
	Steroid cell tumor of the ovary	3	3	100.0	0.0	0.0	0.0
	Brenner tumor	41	39	100.0	0.0	0.0	0.0
Tumors of the breast	Invasive breast carcinoma of no special type	499	485	100.0	0.0	0.0	0.0
	Lobular carcinoma of the breast	192	171	100.0	0.0	0.0	0.0
	Medullary carcinoma of the breast	23	22	100.0	0.0	0.0	0.0
	Tubular carcinoma of the breast	20	11	100.0	0.0	0.0	0.0
	Mucinous carcinoma of the breast	29	24	100.0	0.0	0.0	0.0
	Phyllodes tumor of the breast	50	47	100.0	0.0	0.0	0.0
Tumors of the digestive system	Adenomatous polyp, low-grade dysplasia	50	33	12.1	15.2	24.2	48.5
	Adenomatous polyp, high-grade dysplasia	50	45	15.6	20.0	24.4	40.0
	Adenocarcinoma of the colon	2482	2147	28.9	16.6	22.2	32.4
	Gastric adenocarcinoma, diffuse type	176	150	88.0	6.0	2.0	4.0
	Gastric adenocarcinoma, intestinal type	174	160	79.4	8.1	5.6	6.9
	Gastric adenocarcinoma, mixed type	62	55	85.5	1.8	10.9	1.8
	Adenocarcinoma of the esophagus	83	77	89.6	3.9	5.2	1.3
	Squamous cell carcinoma of the esophagus	75	66	100.0	0.0	0.0	0.0
	Squamous cell carcinoma of the anal canal	89	68	100.0	0.0	0.0	0.0
	Cholangiocarcinoma	50	37	78.4	5.4	5.4	10.8
	Gallbladder adenocarcinoma	31	29	82.8	10.3	0.0	6.9
	Gallbladder Klatskin tumor	41	38	86.8	5.3	5.3	2.6
	Hepatocellular carcinoma	300	285	34.7	3.9	4.2	57.2
	Ductal adenocarcinoma of the pancreas	612	380	98.2	0.5	1.3	0.0
	Pancreatic/ampullary adenocarcinoma	89	61	77.0	1.6	4.9	16.4
	Acinar cell carcinoma of the pancreas	16	15	100.0	0.0	0.0	0.0
	Gastrointestinal stromal tumor (GIST)	50	45	100.0	0.0	0.0	0.0
Tumors of the urinary system	Non-invasive papillary urothelial carcinoma, pTa G2 low grade	177	122	100.0	0.0	0.0	0.0
	Non-invasive papillary urothelial carcinoma, pTa G2 high grade	141	98	100.0	0.0	0.0	0.0
	Non-invasive papillary urothelial carcinoma, pTa G3	219	157	99.4	0.0	0.6	0.0
	Urothelial carcinoma, pT2-4 G3	735	564	99.8	0.0	0.2	0.0
	Squamous cell carcinoma of the bladder	22	21	100.0	0.0	0.0	0.0
	Small cell neuroendocrine carcinoma of the bladder	23	21	100.0	0.0	0.0	0.0
	Sarcomatoid urothelial carcinoma	25	21	100.0	0.0	0.0	0.0
	Urothelial carcinoma of the kidney pelvis	62	60	100.0	0.0	0.0	0.0
	Clear cell renal cell carcinoma	1287	1178	95.8	3.3	0.8	0.0
	Papillary renal cell carcinoma	368	335	98.8	1.2	0.0	0.0
	Clear cell (tubulo)papillary renal cell carcinoma	26	25	100.0	0.0	0.0	0.0
	Chromophobe renal cell carcinoma	170	157	100.0	0.0	0.0	0.0
	Oncocytoma	257	229	100.0	0.0	0.0	0.0
Tumors of the male genital organs	Adenocarcinoma of the prostate, Gleason 3 + 3	83	83	100.0	0.0	0.0	0.0
	Adenocarcinoma of the prostate, Gleason 4 + 4	80	80	100.0	0.0	0.0	0.0
	Adenocarcinoma of the prostate, Gleason 5 + 5	85	85	100.0	0.0	0.0	0.0
	Adenocarcinoma of the prostate (recurrence)	258	258	100.0	0.0	0.0	0.0
	Small cell neuroendocrine carcinoma of the prostate	19	12	100.0	0.0	0.0	0.0
	Seminoma	621	593	100.0	0.0	0.0	0.0
	Embryonal carcinoma of the testis	50	42	97.6	2.4	0.0	0.0
	Leydig cell tumor of the testis	30	30	100.0	0.0	0.0	0.0
	Sertoli cell tumor of the testis	2	2	100.0	0.0	0.0	0.0
	Sex cord stromal tumor of the testis	1	1	100.0	0.0	0.0	0.0
	Spermatocytic tumor of the testis	1	1	100.0	0.0	0.0	0.0
	Yolk sac tumor	50	42	97.6	2.4	0.0	0.0
	Teratoma	50	36	97.2	0.0	0.0	2.8
	Squamous cell carcinoma of the penis	80	80	100.0	0.0	0.0	0.0
Tumors of endocrine organs	Adenoma of the thyroid gland	114	113	100.0	0.0	0.0	0.0
	Papillary thyroid carcinoma	392	369	100.0	0.0	0.0	0.0
	Follicular thyroid carcinoma	154	150	100.0	0.0	0.0	0.0
	Medullary thyroid carcinoma	111	104	100.0	0.0	0.0	0.0
	Parathyroid gland adenoma	43	41	100.0	0.0	0.0	0.0
	Anaplastic thyroid carcinoma	45	41	100.0	0.0	0.0	0.0
	Adrenal cortical adenoma	50	45	100.0	0.0	0.0	0.0
	Adrenal cortical carcinoma	26	22	100.0	0.0	0.0	0.0
	Pheochromocytoma	50	45	100.0	0.0	0.0	0.0
	Appendix, neuroendocrine tumor (NET)	22	14	100.0	0.0	0.0	0.0
	Colorectal, neuroendocrine tumor (NET)	12	11	100.0	0.0	0.0	0.0
	Ileum, neuroendocrine tumor (NET)	49	48	100.0	0.0	0.0	0.0
	Lung, neuroendocrine tumor (NET)	19	18	100.0	0.0	0.0	0.0
	Pancreas, neuroendocrine tumor (NET)	97	80	100.0	0.0	0.0	0.0
	Colorectal, neuroendocrine carcinoma (NEC)	12	11	100.0	0.0	0.0	0.0
	Gallbladder, neuroendocrine carcinoma (NEC)	4	4	100.0	0.0	0.0	0.0
	Pancreas, neuroendocrine carcinoma (NEC)	14	14	100.0	0.0	0.0	0.0
Tumors of hematopoietic and lymphoid tissues	Hodgkin lymphoma	103	76	100.0	0.0	0.0	0.0
	Small lymphocytic lymphoma, B cell type (B-SLL/B-CLL)	50	46	100.0	0.0	0.0	0.0
	Diffuse large B cell lymphoma (DLBCL)	114	106	100.0	0.0	0.0	0.0
	Follicular lymphoma	88	85	100.0	0.0	0.0	0.0
	T-cell Non Hodgkin lymphoma	24	24	100.0	0.0	0.0	0.0
	Mantle cell lymphoma	18	18	100.0	0.0	0.0	0.0
	Marginal zone lymphoma	16	12	100.0	0.0	0.0	0.0
	Diffuse large B-cell lymphoma (DLBCL) in the testis	16	16	100.0	0.0	0.0	0.0
	Burkitt lymphoma	5	3	100.0	0.0	0.0	0.0
Tumors of soft tissue and bone	Tenosynovial giant cell tumor	45	25	100.0	0.0	0.0	0.0
	Granular cell tumor	53	32	100.0	0.0	0.0	0.0
	Leiomyoma	50	47	100.0	0.0	0.0	0.0
	Leiomyosarcoma	87	75	100.0	0.0	0.0	0.0
	Liposarcoma	132	110	100.0	0.0	0.0	0.0
	Malignant peripheral nerve sheath tumor (MPNST)	13	12	100.0	0.0	0.0	0.0
	Myofibrosarcoma	26	26	100.0	0.0	0.0	0.0
	Angiosarcoma	73	59	98.3	0.0	0.0	1.7
	Angiomyolipoma	91	88	100.0	0.0	0.0	0.0
	Dermatofibrosarcoma protuberans	21	17	100.0	0.0	0.0	0.0
	Ganglioneuroma	14	14	100.0	0.0	0.0	0.0
	Kaposi sarcoma	8	6	100.0	0.0	0.0	0.0
	Neurofibroma	117	103	100.0	0.0	0.0	0.0
	Sarcoma, not otherwise specified (NOS)	74	69	100.0	0.0	0.0	0.0
	Paraganglioma	41	41	100.0	0.0	0.0	0.0
	Ewing sarcoma	23	18	100.0	0.0	0.0	0.0
	Rhabdomyosarcoma	6	6	100.0	0.0	0.0	0.0
	Schwannoma	121	113	100.0	0.0	0.0	0.0
	Synovial sarcoma	12	11	100.0	0.0	0.0	0.0
	Osteosarcoma	43	35	100.0	0.0	0.0	0.0
	Chondrosarcoma	38	17	100.0	0.0	0.0	0.0
	Rhabdoid tumor	5	5	100.0	0.0	0.0	0.0

**Table 2:** FABP1 immunostaining and tumor phenotype in colon cancers

				FABP1 IHC result				
			n	Negative (%)	Weak (%)	Moderate (%)	Strong (%)	*P*
Colon adenocarcinoma (all cancers)	Primary Tumor	pT1	78	29.5	14.1	17.9	38.5	0.5175
		pT2	403	28.8	16.9	23.1	31.3	
		pT3	1144	27.6	15.7	22.9	33.7	
		pT4	413	32	18.4	20.3	29.3	
	Grade	1	5	20	0	40	40	< 0.0001
		2	523	26	17	22.9	34.1	
		3	65	64.6	12.3	7.7	15.4	
	Regional lymph nodes	pN0	1073	28.2	17.6	22.4	31.8	0.6035
		pN +	956	29.5	15.5	22.2	32.8	
	Lymphatic invasion	L0	659	31.6	15.2	21.4	31.9	0.3808
		L1	1348	28.1	17.2	22.1	32.6	
	Tumor localization	Left colon	1112	25.4	15.9	23.4	35.3	< 0.0001
		Right colon	417	36.9	18.2	20.4	24.5	
	MMR status	Defective	84	56	21.4	11.9	10.7	< 0.0001
		Proficient	1067	25	16.2	23.7	35.1	
	RAS mutation status	Mutated	325	26.8	18.8	23.4	31.1	0.058
		Wild type	414	24.2	13	23.9	38.9	
	BRAF mutation status	Mutated	14	78.6	7.1	7.1	7.1	0.001
		Wild type	90	23.3	15.6	27.8	33.3	
Colon adenocarcinoma (microsatellite stable cancers)	Primary TUMOR	pT1	41	34.1	17.1	22	26.8	0.7281
		pT2	221	24.4	18.1	24.4	33	
		pT3	587	23.2	15.3	24.5	37	
		pT4	207	28	16.4	21.3	34.3	
	Grade	1	0	-	-	-	-	
		2	26	23.1	15.4	38.5	23.1	0.3235
		3	4	25	50	25	0	
	Regional lymph nodes	pN0	550	25.3	16	26	32.7	0.2234
		pN +	498	24.7	16.3	21.3	37.8	
	Lymphatic invasion	L0	423	26.5	13.9	25.5	34	0.2894
		L1	602	24.6	17.4	22.1	35.9	
	Tumor localization	Left colon	819	23	16.1	24.1	36.9	0.0372
		Right colon	243	31.7	16	23	29.2	
	RAS mutation status	Mutated	262	24.4	17.9	23.7	34	0.0631
		Wild type	326	19.3	12.9	24.8	42.9	
	BRAF mutation status	Mutated	6	50	16.7	16.7	16.7	0.4871
		Wild type	71	21.1	16.9	28.2	33.8	
Colon adenocarcinoma (microsatellite instable cancers)	Primary tumor	pT1	6	66.7	0	0	33.3	0.1955
		pT2	19	57.9	21.1	5.3	15.8	
		pT3	40	57.5	25	15	2.5	
		pT4	19	47.4	21.1	15.8	15.8	
	Regional lymph nodes	pN0	54	55.6	22.2	9.3	13	0.3852
		pN +	28	57.1	21.4	17.9	3.6	
	Lymphatic invasion	L0	37	73	16.2	2.7	8.1	0.0203
		L1	45	44.4	26.7	20	8.9	
	Tumor localization	Left colon	36	41.7	16.7	19.4	22.2	0.0023
	Lymphatic invasion	Right colon	48	66.7	25	6.3	2.1	
	RAS mutation status	Mutated	8	37.5	12.5	37.5	12.5	0.5705
	Tumor localization BRAF mutation status	Wild type	21	57.1	19	14.3	9.5	
		Mutated	5	100	0	0	0	0.1174
	RAS mutation status	Wild type	9	44.4	11.1	33.3	11.1	
Hepatocellular carcinoma	Primary tumor	pT1	90	20.0	5.6	4.4	70.0	0.0002
		pT2	100	37.0	1.0	3.0	59.0	
		pT3	62	51.6	4.8	8.1	50.0	
	Regional lymph nodes	pN0	81	40.7	6.2	3.7	49.4	0.0042
		pN +	44	72.7	2.3	4.5	20.5	
	Grade	G1	45	13.3	6.7	6.7	73.3	0.0728
		G2	160	36.9	3.1	4.4	55.6	
		G3	56	30.4	1.8	3.6	64.3	
	Histology	NOS	138	4.3	2.2	5.1	88.4	0.0994
		Carcinosarcoma	1	0.0	0.0	0.0	100.0	
		Clear cell	4	0.0	0.0	0.0	100.0	
		Lipid-rich	3	33.3	0.0	0.0	66.7	
		Lymphocyte-rich	2	0.0	0.0	0.0	100.0	
		Scirrhous	9	33.3	22.2	11.1	33.3	
		Steatohepatitic	21	0.0	0.0	0.0	100.0	
	Growth pattern	Solid	64	3.1	1.6	4.7	90.6	0.0645
		Trabecular	77	2.6	3.9	2.6	90.9	
		Macrotrabecular	10	10.0	10.0	0.0	80.0	
		Pseudoglandular	26	19.2	0.0	11.5	69.2	
	Fatty change	No	142	6.3	3.5	4.9	85.2	0.2848
		Yes	37	2.7	0.0	2.7	94.6	
	Gender	Male	203	27.6	3.9	3.4	65.0	0.0002
		Female	81	53.1	3.7	6.2	37.0	
	Age (yrs)	≤ 50	27	40.7	11.1	3.7	44.4	0.0648
		51–60	55	34.5	0.0	5.5	60.0	
		61–70	93	32.3	4.3	4.3	59.1	
		71–80	91	35.2	4.4	3.3	57.1	
		> 80	19	36.8	0.0	5.3	57.9	

## Discussion

Considering the large scale of our study, emphasis was placed on the appropriate validation of our FABP1 immunohistochemistry assay. Based on recommendations of the International Working Group for Antibody Validation (IWGAV), we compared our FABP1 staining data with expression data obtained by another independent method [26]. Normal tissue RNA expression data derived from three different publicly accessible databases [27–30] were therefore compared with immunostaining results in 76 different normal tissue categories. This broad range of tissues is likely to contain most proteins that are normally expressed at relevant levels in cells of adult humans and should therefore enable the detection of most undesired cross-reactivities of tested antibodies. Specificity of our assay was supported by the limitation of FABP1 immunostaining to kidney, liver, and the intestine. These are the only organs for which significant FABP1 RNA expression had been described.

**Fig. 4:**
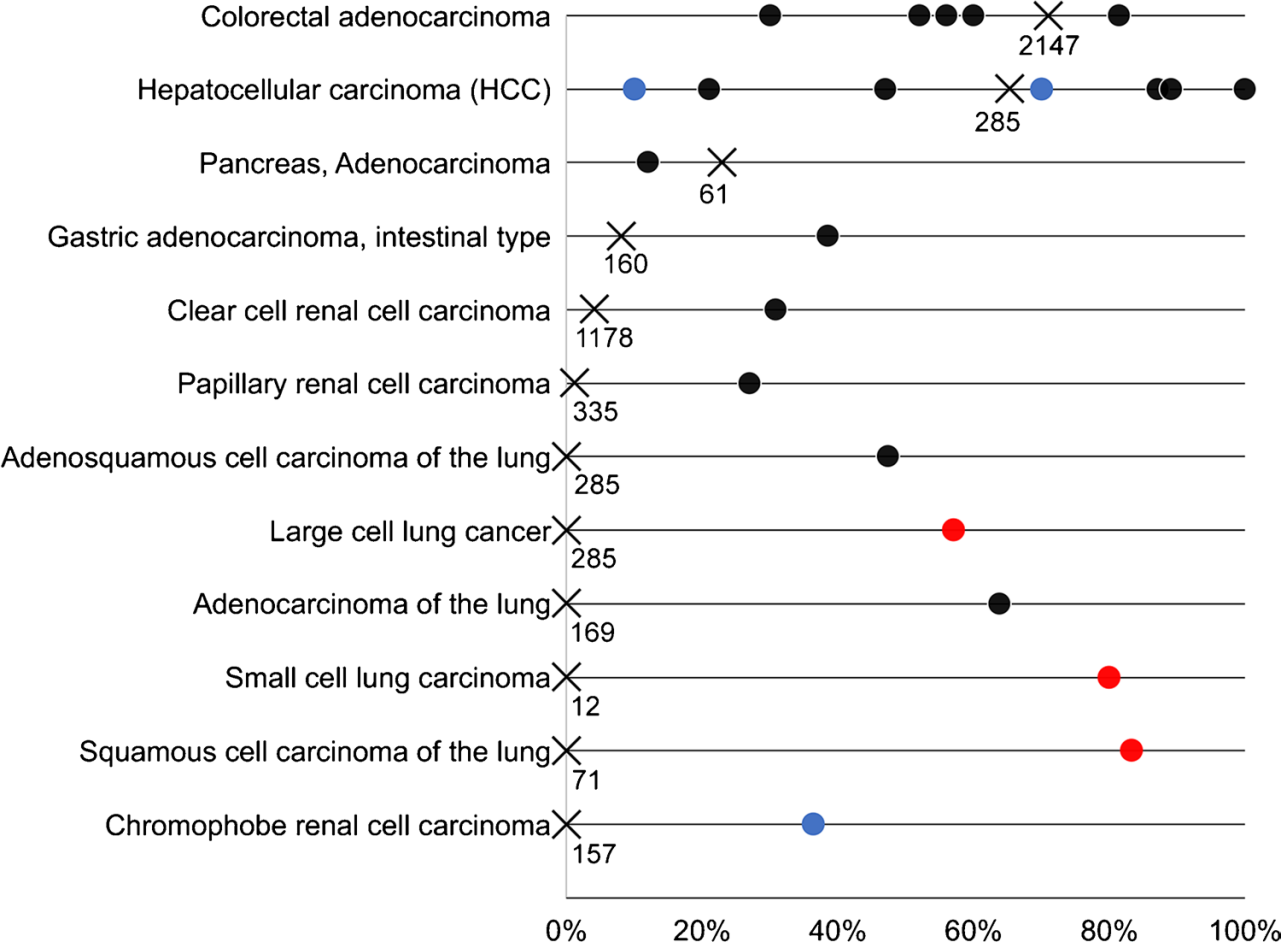
Fraction of FABP1 positivity per tumor type from previous literature. Colors of the dots represent the numbers of analyzed tumors in these studies: red, *n* = 1–10; blue, *n* = 11–25; black, *n* > 25. X = results of this study, with numbers indicating the sample size

Our data provide a comprehensive overview on the prevalence and intensity of FABP1 immunostaining across a large variety of human tumor entities. The findings demonstrate that FABP1 expression occurs at highest frequency (65–80%) in hepatocellular carcinomas and colorectal adenocarcinomas, at lower frequency (35%) in mucinous carcinoma of the ovary and in other adenocarcinomas of the digestive tract (10–25%), and only rarely (< 5%) in a limited number of other tumor types. These data not only expand the existing literature but also clarify existing findings which in part are highly discordant with our data. A total of 15 previous studies have reported IHC findings on FABP1 in 12 different tumor entities (results summarized in Fig. 4). While multiple studies describe FABP1 expression frequencies that are in the range of our findings in hepatocellular carcinomas [31–33], colorectal adenocarcinomas [16, 34], pancreatic adenocarcinomas [19], and gastric adenocarcinoma [17], we were unable to detect any FABP1-positive cases among 169 adenocarcinomas of the lung, 12 small cell carcinomas of the lung, and 157 chromophobe carcinomas of the kidney. For all these entities, others have described substantial fractions of FABP1-positive cases [14, 18]. Absence of FABP1 expression in lung and kidney cancer is also supported by RNA expression studies summarized in the ICGC/TCGA databases (https://www.cancer.gov/about-nci/organization/ccg/research/structural-genomics/tcga) and The Human Protein Atlas [30].

Our comprehensive set of data on FABP1 immunostaining in tumors suggests a potential diagnostic utility of FABP1 immunohistochemistry in surgical pathology. While it is obvious from our data that a positive FABP1 immunostaining in a metastatic tissue of unknown origin would pinpoint towards the liver or the gastrointestinal tract as the most likely sites of cancer origin, the highest diagnostic utility may be derived from the constant absence of FABP1 immunostaining in 169 analyzed adenocarcinomas of the lung. As the lung is a common site of metastases, the distinction of primary lung adenocarcinoma from metastatic adenocarcinoma is a frequent diagnostic problem which has high therapeutic implications. A potential utility for this application is particularly supported by the absence of FABP1 staining in 20 pulmonary adenocarcinomas for which cytokeratin 20, SATB2, and/or villin positivity had suggested a possible intestinal/enteric differentiation. A low likelihood of pulmonary adenocarcinomas to become FABP1 positive is also supported by the complete lack of FABP1 RNA expression in 510 pulmonary adenocarcinomas described in the TCGA Pan Cancer Atlas database (https://www.cancer.gov/about-nci/organization/ccg/research/structural-genomics/tcga). A positive FABP1 immunostaining in an adenocarcinoma in the lung may therefore be highly suggestive of an extra-pulmonary tumor origin and favor a metastasis derived from a colorectal cancer or another cancer of the gastrointestinal tract. However, considering that only 71% of our colorectal adenocarcinomas were FABP1 positive and the even lower frequency of FABP1 positivity in other gastrointestinal adenocarcinomas, a negative FABP1 staining cannot serve as evidence for a pulmonary origin of an adenocarcinoma in the lung. Moreover, in case of an adenocarcinoma in the pancreas, FABP1 positivity would argue in favor of a carcinoma derived from the ampulla of Vater (23% positive) and against a ductal adenocarcinoma (1.8% positive). Loss of FABP1 expression in a hepatic tumor has been described as a feature of hepatocellular adenoma [35, 36]. However, our data show that 50–70% of advanced and metastatic hepatocellular carcinomas and up to 20% of low-stage and grade carcinomas may be FABP1 negative. These observations are in line with earlier reports [31, 37, 38] suggesting that a lack of FABP1 staining should be interpreted with care to avoid misdiagnosing a well-differentiated hepatocellular carcinoma as hepatocellular adenoma.

It is of note that FABP1 expression in normal and neoplastic tissues is usually either high or absent. In immunohistochemical analysis, this often results in such an abundant staining reaction that bound antibody can also be seen in the vicinity of FABP1-expressing cells. Such a spill-over of FABP1 protein may either be caused by some physiologic intravital diffusion of the highly abundant FABP1 protein or reflect an ischemia-induced artifact caused by autolytic cell damage occurring between removal of the tissue from the patient and completed tissue fixation. Such “contamination artifacts” must be considered if metastatic tissue is seen in biopsies from the liver because they can lead to questionable staining or false positivity.

The successful analysis of more than 2000 colorectal adenocarcinomas enabled us to analyze the relationship between FABP1 expression, tumor phenotype, and molecular data in this tumor entity. That low FABP1 expression was strongly linked to high-grade, MSI, and right-sided tumor location but unrelated to pT and pN stage is consistent with the results of two earlier studies. In a study on 695 colorectal carcinomas, Wood et al. [39] described a strong link of low FABP1 with MSI and high histologic grade but also failed to find significant associations with advanced stage or patient survival. Lawrie et al. [15] analyzed 249 colorectal adenocarcinomas and found a relationship between low FABP1 and high grade but did not see associations with tumor stage. The mechanism causing low FABP1 expression in colorectal adenocarcinomas with MSI is unclear. Wood et al. [39] suggested a possible role of PPARγ and the interferon γ pathway. It also appears possible that one or several genes that are required for FABP1 expression are inactivated by accumulating mutations in MSI cancers. Silencing of FABP1 expression by specific molecular events is not uncommon. In hepatocellular adenomas, efficient silencing of FABP1 can be caused by biallelic inactivation of hepatocyte nuclear factor 1α (HNF1A) which occurs in 35–40% of cases [40]. That reduced FABP1 expression was driven by high grade—a feature that is commonly related to MSI— and not by MSI in our multivariate analysis may suggest, however, that FABP1 expression loss is merely an indicator of poor differentiation and may not have further biological meanings. With respect to molecular mechanisms for FABP1 inactivation, it is also remarkable that FABP1 expression is virtually absent in kidney cancers, although the protein is abundantly seen in the normal kidney.

In summary, our data show that FABP1 expression has high tumor specificity and preferentially occurs in hepatocellular carcinomas, colorectal carcinomas, mucinous ovarian cancer, and other gastrointestinal adenocarcinomas. As FABP1 expression is virtually absent in adenocarcinomas of the lung, FABP1 immunohistochemistry might be most helpful for its distinction from metastatic adenocarcinoma to the lung.

## Supplementary Information

Below is the link to the electronic supplementary material.

Supplementary file1 (PPTX 38563 KB)

Supplementary file2 (XLSX 11 KB)

Supplementary file3 (XLSX 10 KB)

## Data Availability

All data generated or analyzed during this study are included in this published article. Raw data are available upon reasonable request.
