# Supplementary material for: FABP1 expression in human tumors: a tissue microarray study on 17,071 tumors
Source: Virchows Arch. 2022 Aug 11;481(6):945–61. doi: 10.1007/s00428-022-03394-5 (PMC9734244; doi:10.1007/s00428-022-03394-5)
Supplement: Supplementary file 1 — Supplementary file1 (PPTX 38563 KB) [file 428_2022_3394_MOESM1_ESM.pptx]

## Slide 1
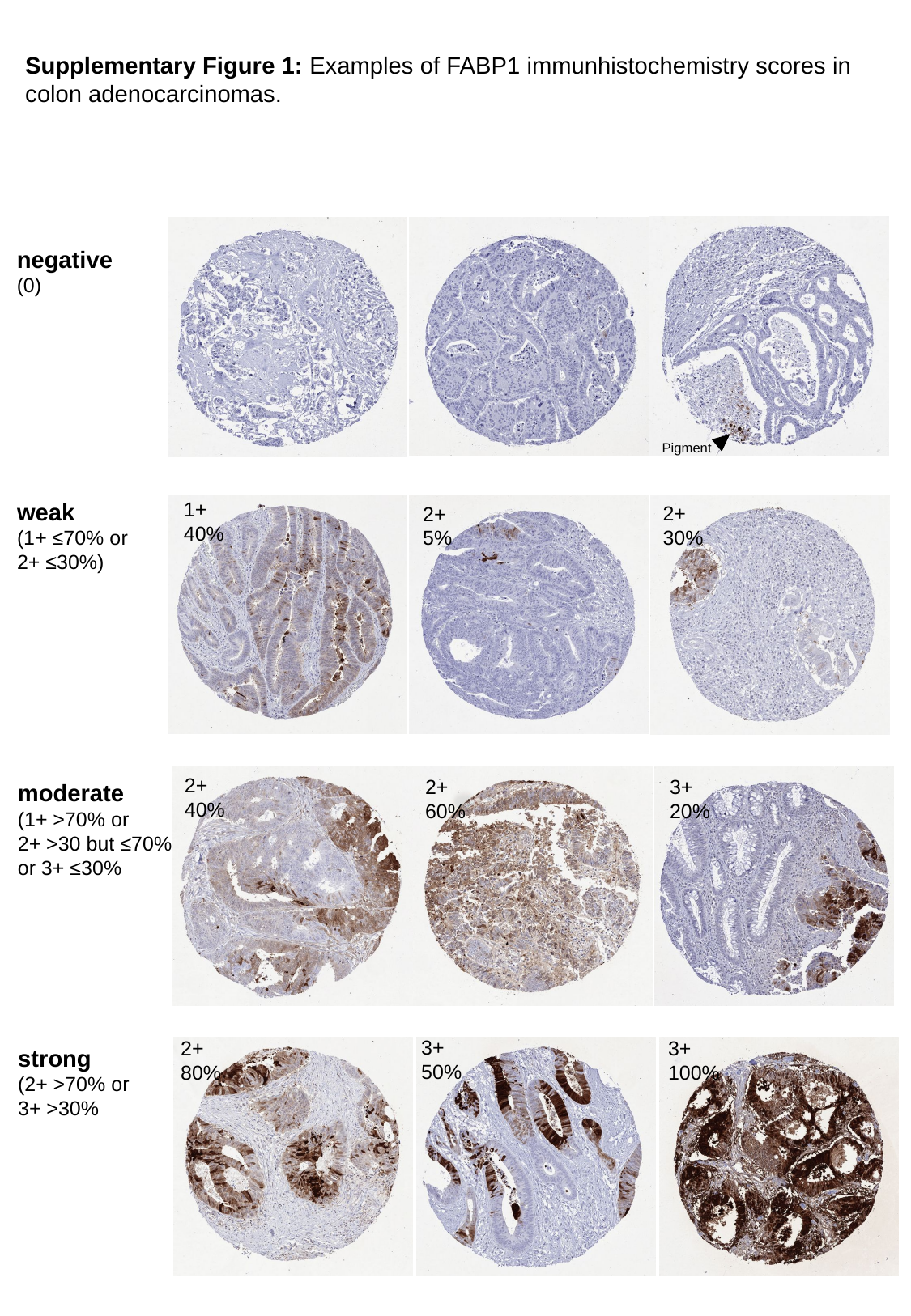

Supplementary Figure 1: Examples of FABP1 immunhistochemistry scores in colon adenocarcinomas.
negative
(0)
Pigment
weak
(1+ ≤70% or
2+ ≤30%)
1+
40%
2+
30%
2+
5%
2+
40%
2+
60%
3+
20%
moderate
(1+ >70% or
2+ >30 but ≤70%
or 3+ ≤30%
3+
50%
3+
100%
2+
80%
strong
(2+ >70% or
3+ >30%
